# Supplementary material for: Light therapy for sleep disturbance comorbid depression in relation to neural circuits and interactive hormones—A systematic review
Source: PLoS One. 2023 Sep 28;18(9):e0286569. doi: 10.1371/journal.pone.0286569 (PMC10538739; doi:10.1371/journal.pone.0286569)
Supplement: S1 Fig — The high-definition version of the figures in this article. (PDF) [file pone.0286569.s001.pdf]

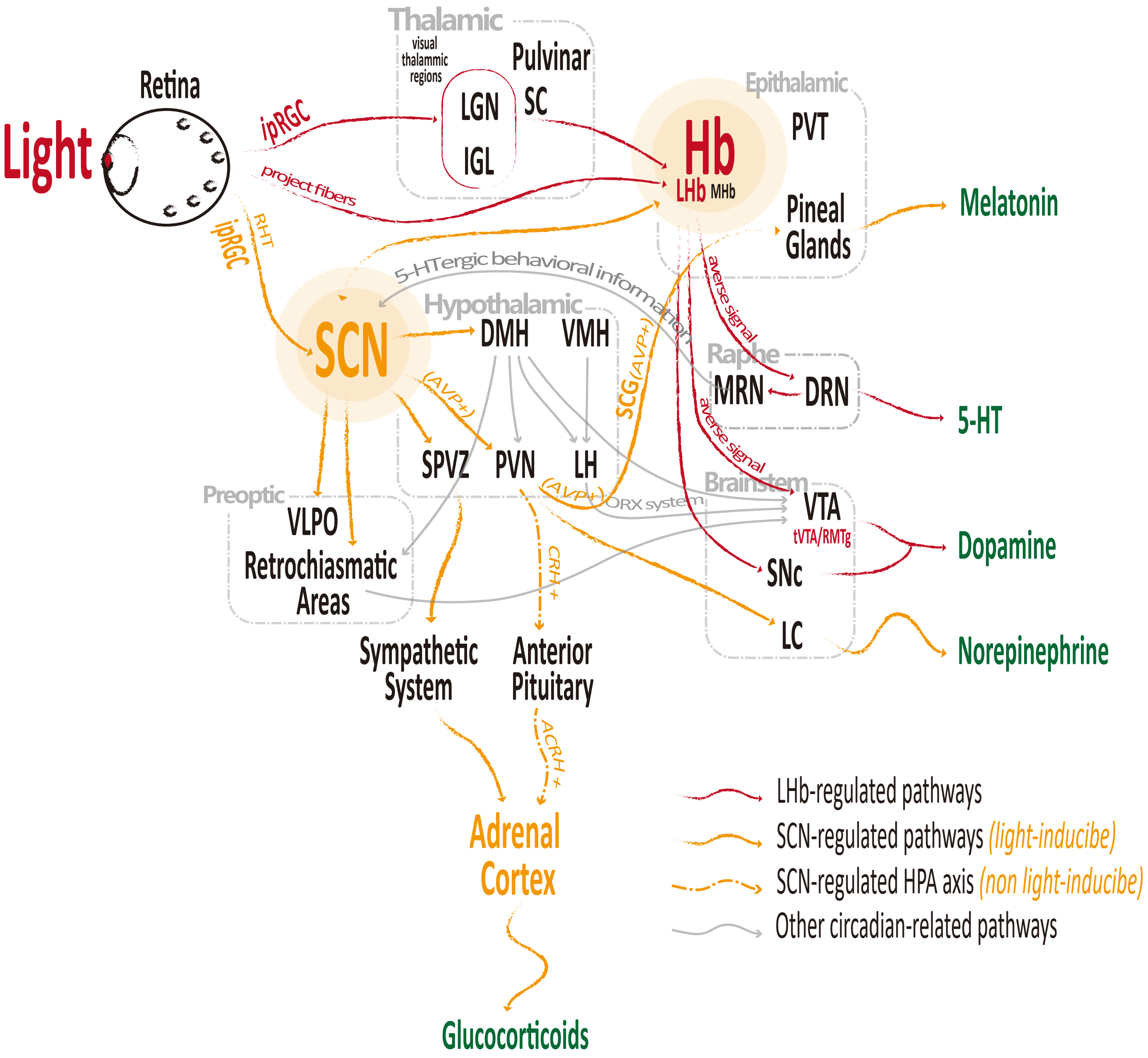


Fig. 2 Light-responsive pathways for circadian regulation

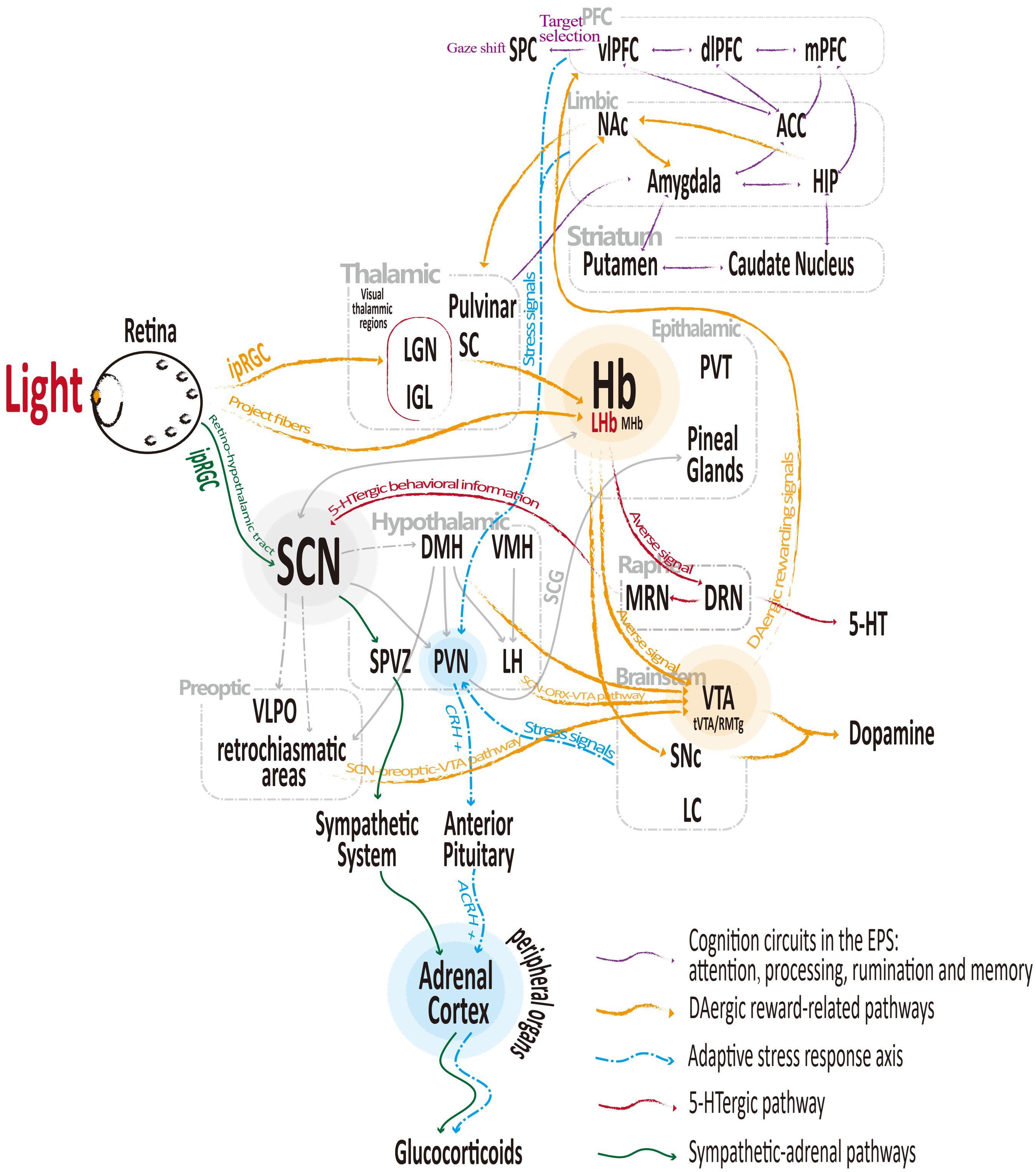


Fig. 3 Light-responsive pathways for emotional regulation

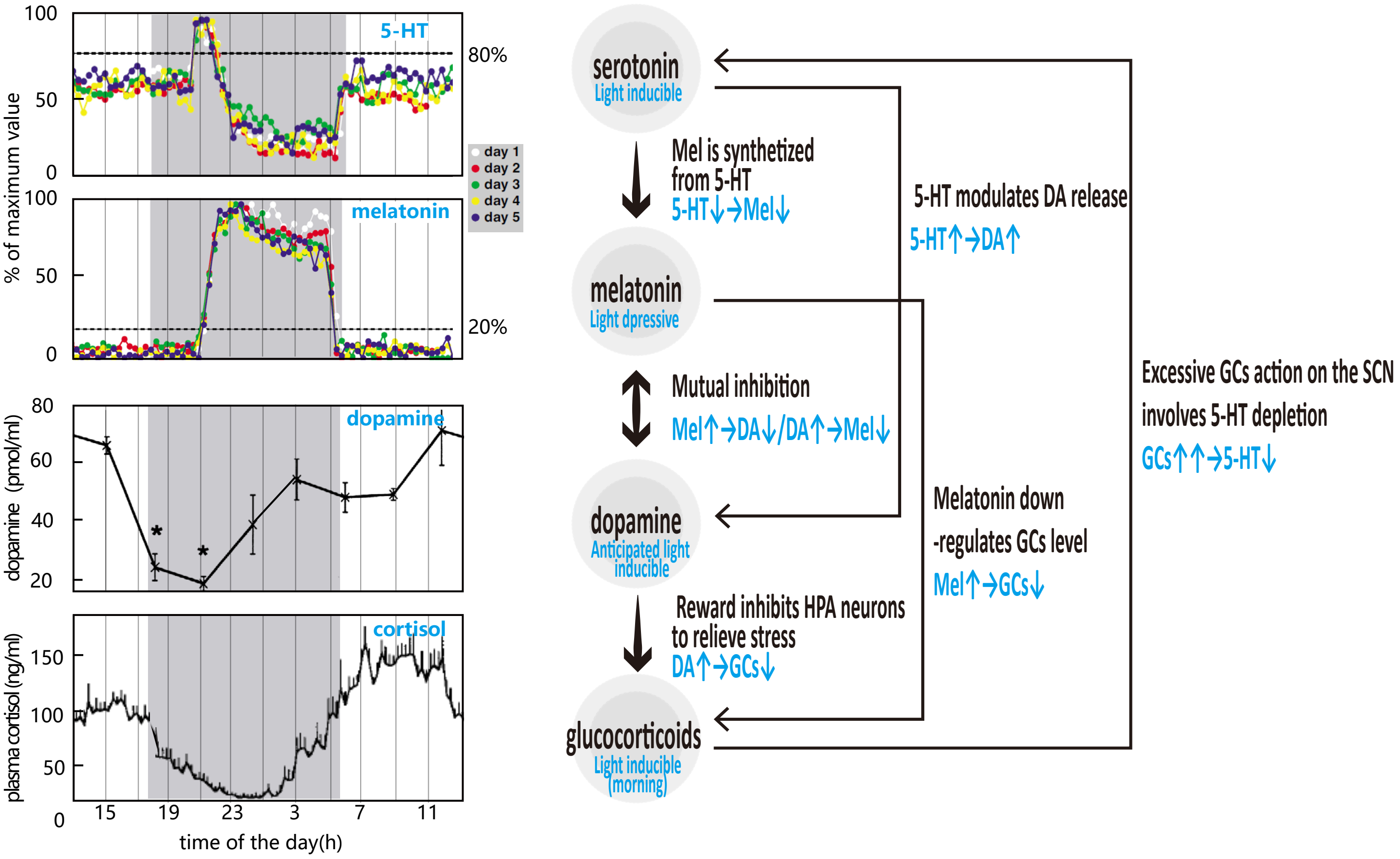


Fig. 4 Hormone daily variation (left) and hormonal interactions (right)

(5-HT and melatonin variation is re-drew according to literature 44, DA variation is re-drew according to literature 60, and cortisol variation is re-drew according to literature 53)
